# Potential lncRNA Biomarkers for HBV-Related Hepatocellular Carcinoma Diagnosis Revealed by Analysis on Coexpression Network

**DOI:** 10.1155/2021/9972011

**Published:** 2021-10-15

**Authors:** Shunqiang Nong, Xiaohao Chen, Zechen Wang, Guidan Xu, Wujun Wei, Bin Peng, Lü Zhou, Liuzhi Wei, Jingjie Zhao, Qiuju Wei, Yibin Deng, Lingzhang Meng

**Affiliations:** ^1^Center for Systemic Inflammation Research (CSIR), School of Preclinical Medicine, Youjiang Medical University for Nationalities, Baise City, Guangxi Province, China; ^2^Clinic Medicine Research Center of Hepatobiliary Diseases, The Affiliated Hospital of Youjiang Medical University for Nationalities, Baise, 533000 Guangxi Province, China; ^3^Department of Infectious Diseases, The Affiliated Hospital of Youjiang Medical University for Nationalities, Baise, 533000 Guangxi Province, China; ^4^Centre for Medical Laboratory Science, The Affiliated Hospital of Youjiang Medical University for Nationalities, Baise, 533000 Guangxi, China; ^5^Life Science and Clinical Research Center, The Affiliated Hospital of Youjiang Medical University for Nationalities, Baise City, Guangxi Province, China

## Abstract

**Background:**

Increasing evidence demonstrated that long noncoding RNA (lncRNA) could affect inflammatory tumor immune microenvironment by modulating gene expression and could be used as a biomarker for HBC-related hepatocellular carcinoma (HCC) but still needs further research. The aim of the present study was to determine an lncRNA signature for the diagnosis of HBV-related HCC.

**Methods:**

HBV-related HCC expression profiles (GSE55092, GSE19665, and GSE84402) were abstracted from the GEO (Gene Expression Omnibus) data resource, and R package limma and RobustRankAggreg were employed to identify common differentially expressed genes (DEGs). Using machine learning, optimal diagnostic lncRNA molecular markers for HBV-related HCC were identified. The expression of candidate lncRNAs was cross-validated in GSE121248, and an ROC (receiver operating characteristic) curve of lncRNA biomarkers was carried out. Additionally, a coexpression network and functional annotation was built, after which a PPI (protein-protein interaction) network along with module analysis were conducted with the Cytoscape open source software.

**Result:**

A total of 38 DElncRNAs and 543 DEmRNAs were identified with a fold change larger than 2.0 and a *P* value < 0.05. By machine learning, AL356056.2, AL445524.1, TRIM52-AS1, AC093642.1, EHMT2-AS1, AC003991.1, AC008040.1, LINC00844, and LINC01018 were screened out as optional diagnostic lncRNA biosignatures for HBV-related HCC. The AUC (areas under the curve) of the SVM (support vector machine) model and random forest model were 0.957 and 0.904, respectively, and the specificity and sensitivity were 95.7 and 100% and 94.3 and 86.5%, respectively. The results of functional enrichment analysis showed that the integrated coexpressed DEmRNAs shared common cascades in the p53 signaling pathway, retinol metabolism, PI3K-Akt signaling cascade, and chemical carcinogenesis. The integrated DEmRNA PPI network complex was found to be comprised of 87 nodes, and two vital modules with a high degree were selected with the MCODE app.

**Conclusion:**

The present study identified nine potential diagnostic biomarkers for HBV-related HCC, all of which could potentially modulated gene expression related to inflammatory conditions in the tumor immune microenvironment. The functional annotation of the target DEmRNAs yielded novel evidence in evaluating the precise functions of lncRNA in HBV-related HCC.

## 1. Introduction

Hepatocellular carcinoma (HCC) is a primary tumor that originates from hepatocytes, which is one of the most frequent malignant tumors and ranks sixth in incidence and fourth in mortality globally [1]. Chronic hepatitis B virus (HBV) infection constitutes the primary risk factor in most high-risk HCC areas. Multiple studies have documented that HBV participates in the carcinogenesis and invasion as well as metastasis of liver cells, playing a crucial role in the onset and development of liver cancer [2–4]. Given the lack of distinct clinical manifestations, most diagnosed HBV-linked HCC patients are in the advanced stage and have a poor prognosis. Although some serum biomarkers for early diagnosis have been reported in the literature, the results were not very satisfactory [5–7]. Therefore, finding new molecular markers for the early diagnosis, treatment, and prognostic evaluation of HBV-related HCC is urgently required.

Long noncoding RNAs (lncRNAs) constitute a large group of transcribed RNA biomolecules that are longer than 200 nucleotides, but their protein-coding ability is limited since they lack functional open readings [8]. lncRNAs modulate gene expression of posttranslational, translational, posttranscriptional, and transcriptional as well as epigenetic levels in diverse ways [9, 10]. Moreover, lncRNAs have been documented to participate actively in the modulation of diverse aspects of tumor progress consisting of growth and metastasis, as well as recurrence [11, 12]. For example, HULC, a well-explored lncRNA that is associated with HBV infection along with HCC tumor growth, has been reported to be upregulated in HCC and is linked to its grades, metastasis, and drug resistance [11]. Upregulated-lncRNA ZEB1-AS1 is correlated with tumor growth along with metastasis in HCC via modulating the expression of epithelial-mesenchymal transition-mediated markers, and patients with high expressions of ZEB1-AS1 exhibit high metastatic relapse, as well as dismal survival [13]. lncRNA MALAT1 is responsible for proliferation, migration, and infiltration, as well as apoptosis through its interactions with miR-200a in hypoxic Hep3B cells. Evidence of lncRNA dysregulation, along with the occurrence and development of HCC, has been gradually increasing. However, studies on HBV-related HCC are relatively limited.

Herein, three microarray expression data of a large number of HBV-related HCC patients were abstracted from the NCBI-GEO data resource, and the DElncRNAs (differentially expressed lncRNAs), as well as DEmRNAs (differentially expressed mRNAs), between HBV-related HCC and nontumor tissues were screened. Feature selection along with classification models was conducted to identify optimal lncRNAs with diagnostic value for HBV-related HCC. GO (Gene Ontology) and KEGG (Kyoto Encyclopedia of Genes and Genomes) analyses, as well as the PPI (protein-protein interaction) and hub gene identification, were also performed. Accordingly, this study provided novel biomarkers for diagnosis and identified promising treatment targets for the treating HBV-related HCC.

## 2. Methods and Materials

### 2.1. Microarray Data Analysis

The NCBI-GEO data resource (https://www.ncbi.nlm.nih.gov/geo/) is a free repository of microarray gene patterns and next-generation sequencing, from which HBV-related hepatocellular carcinomas and nontumor or neighboring tissue gene expression profiles of GSE55092, GSE19665, and GSE84402 were abstracted. The three microarray data were all based on GPL570 platforms (HG-U133_Plus_2) and the Affymetrix Human Genome U133 Plus 2.0 Array.GSE55092, GSE19665, and GSE84402 contained 91, 5, and 13 cases of nontumor tissues from hepatitis B patients, respectively, and 49, 5, and 13 cases of HBV-associated HCC tissues, respectively. In order to acquire the international standard gene name, the probe was reannotated to determine the expression levels of lncRNA, as well as mRNA potentially related to HBV-related HCC. Briefly, the chip sequence file was first downloaded in FASTA format from the Affymetrix web page (www. http://affymetrix.com). Second, we aligned the probe sets of the HG-U133_Plus_2.0 array to the human genome (GRCh38), as well as the lncRNA gene sequence from GENCODE (https://www.gencodegenes.org/) (release 30) using the SeqMap tool with no mismatches. Finally, 4,971 probes were matched with 3,750 lncRNAs, and 31,475 probes were matched with 15,435 mRNAs in the HG-U133 Plus 2.0 microarray. Subsequently, the gene expression data that was subjected to log2 transformation was normalized between the “Arrays” function in the R limma package (http://www.bioconductor.org/).

### 2.2. Screening for Differentially Expressed mRNAs and lncRNAs

The DElncRNAs along with the DEmRNAs between HBV-linked HCC and nontumor tissues were computed through the limma R package V3.5.2 in R software in each dataset. To avoid missing those that are rare but important in the regulation of pathways, we set the threshold of the expression of DElncRNAs, as well as DEmRNAs, to *P* < 0.05 and ∣log2FC | >1. Afterwards, the RobustRankAggreg (RRA) R package was used to integrate and analyze the three gene lists that were subsequently sorted via the logFC value. The package RobustRankAggreg (RRA) is usually used for comparing sequenced gene lists based on reviewing each gene's ranking position (in case that each gene is randomly arranged). We exported and saved the lists of remarkably upregulated, as well as downregulated, genes as Excel files. After that, the hierarchical clustering of DEmRNAs and DElncRNAs was carried out with the R "heatmap” package.

### 2.3. Identifying the Optimal Diagnostic lncRNA Molecular Markers for HBV-Related HCC

The random forest analysis was employed to perform the feature selection procedures which were conducted to determine the optimal diagnostic lncRNA biosignatures for HBV-related HCC. Additionally, the importance value of every DElncRNA was ranked as per the MDG (Mean Decrease Gini). The optimal number of features was then determined by subsequently adding one DElncRNA at a time in a top-down forward-wrapper manner. Through applying the SVM (support vector machine) at each increment, we explored the accuracy and uncovered the optimal diagnostic lncRNA molecular markers for HBV-related HCC. Selection of the optimal DElncRNAs in HBV-related HCC, as well as nontumor tissue, was then performed. The random forest model and the SVM model were established using the randomForest package and the e1071 package, respectively.

### 2.4. Cross-Validation

Furthermore, another microarray dataset was collected from the GEO database under accession number GSE121248 (platform: GPL570 [HG-U133_Plus_2] Affymetrix Human Genome U133 Plus 2.0 Array) to serve as the validation set. GSE121248 contained 37 cases of nontumor tissues from hepatitis B patients as well as 70 cases of HBV-linked HCC tissues. The diagnostic ability of each model and lncRNA molecular markers was explored by the ROC (receiver operating characteristic), AUC (area under curve), sensitivity, and specificity in the validation set.

### 2.5. DEmRNA-DElncRNA Coexpressed Analysis

The coexpression network searches for the core modulated genes in the network and locates the functions along with the signal transduction cascades in which lncRNA participates via the genes it modulates. Here, the coexpression network evidently exhibited the association of the lncRNAs with the genes and the overall cross-talk, facilitating the discovery of the modulatory correlation of the lncRNAs with the genes, as well as the evaluation of its potential functional impacts. The correlation between DElncRNAs and DEmRNAs was assessed by the pairwise Pearson correlation coefficient. Besides, the threshold for DElncRNA-DEmRNA coexpression pairs was adjusted to *P* < 0.05 and ∣*r* | >0.5. Afterwards, the HBV-related HCC-specific DElncRNA-DEmRNA coexpression network was generated using the Cytoscape platform (http://cytoscape.org).

### 2.6. Functional and Signal Pathway Enrichment Analysis

The DAVID version 6.8 data resource (http://david.ncifcrf.gov) is a web-accessible program for integrating biological data that consists of analysis tools, which provide an exhaustive set of functional annotation data of extensive lists of proteins or genes for researchers to comprehend the biological features. In order to reveal the biological roles along with the potential cascades of the genes coexpressed using optimal lncRNAs with a diagnostic value for HBV-related HCC, GO classification and KEGG pathway enrichment were conducted using DAVID, in which *P* < 0.05 signified significant difference.

### 2.7. Protein-Protein Interaction Network and Module Analysis

The STRING online data resource (https://string-db.org/) was employed to develop the PPI network for screening genes, where the interaction score was set to >0.4. Cytoscape was employed to visualize the results, and the MCODE (Molecular Complex Detection) plug-in was employed to screen the modules or clusters of the PPI network. The MCODE parameters were set by default, and the KEGG as well as GO assessments of DEGs in modules were utilized in their investigation using DAVID, in which *P* < 0.05 was set at the cut-off criterion.

## 3. Results

### 3.1. Differentially Expressed lncRNAs and mRNAs

**Figure 1:**
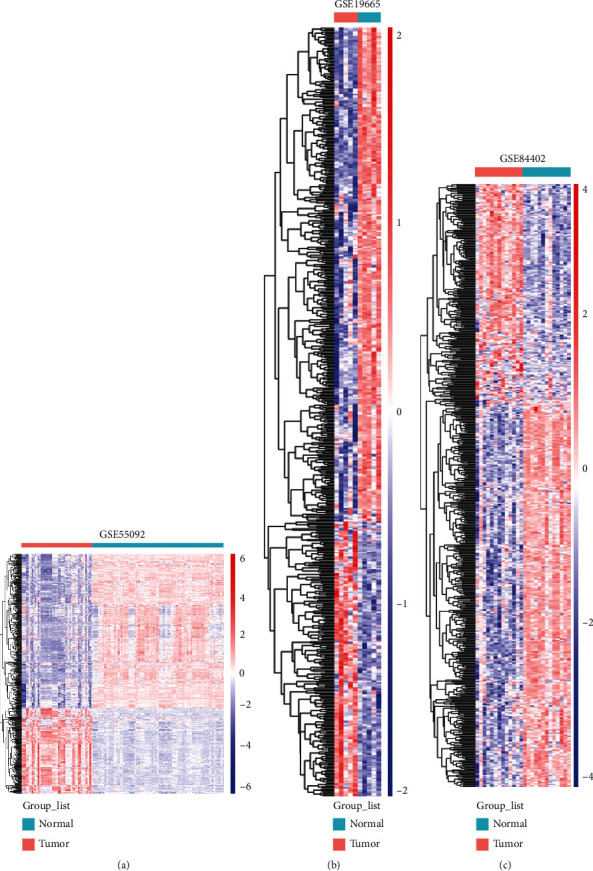
Heat maps of the consensus differentially expressed RNAs in GSE55092, GSE19665, and GSE84402. Green and red represent normal and tumor samples, respectively.

Before data analysis, we performed principal component analysis (PCA) for quality control (Supplementary figure (available here)); the PCA plots showed that the three datasets explored in this study exhibited distinguished lncRNA profiles between normal and tumor biopsies. Three HBV-linked HCC gene expression patterns were abstracted from the GEO data resource. Afterward, we normalized the gene expression data, and the DEGs were determined with the limma package of R. Using *P* < 0.05 and ∣logFC | >1 as cut-off criteria, 103, 333, and 158 differentially expressed lncRNAs (DElncRNAs) and 1182, 2147, and 1579 differentially expressed mRNAs (DEmRNAs) were extracted from the expression profile datasets GSE55092, GSE19665, and GSE84402, respectively, presented in heat map plots (Figure 1). On the basis of the integration along with the analysis of RRA, 38 DElncRNAs, including 25 upregulated and 13 downregulated lncRNAs, as well as 541 DEmRNAs, including 348 upregulated and 195 downregulated mRNAs, of invariably expressed genes were uncovered from the three profile cohorts.

### 3.2. Optimal Diagnostic lncRNA Biosignatures for HBV-Related HCC

**Figure 2:**
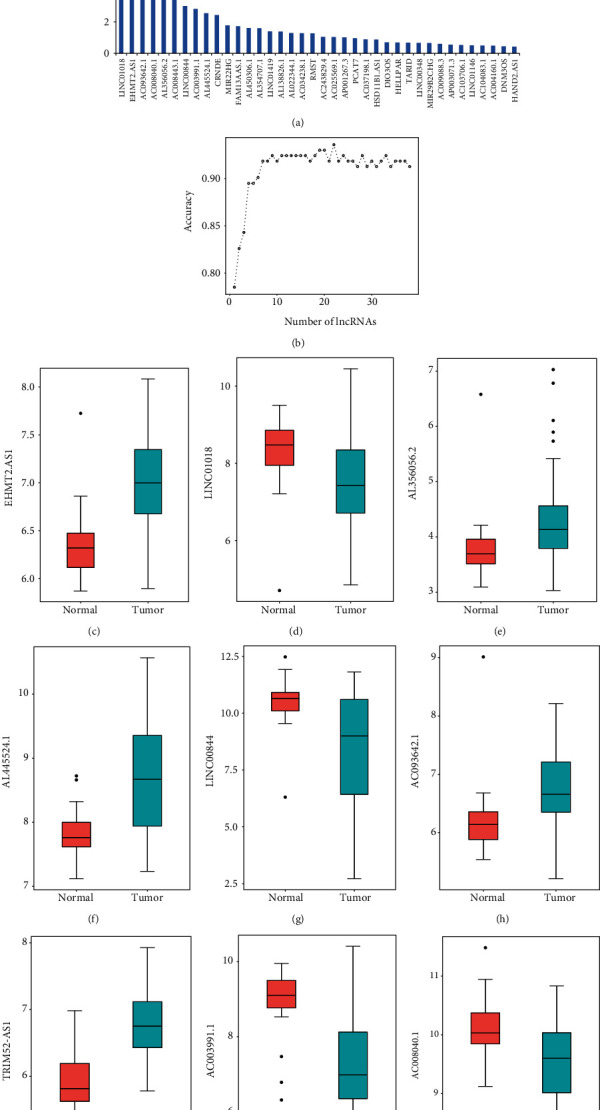
Determination of the prospective lncRNA biosignatures for HBV-related HCC. (a) The importance value of every DElncRNA was ranked as per the Mean Decrease Gini through the random decision forest. (b) The variance rate of classification performance when increasing numbers of the predictive DElncRNAs. Box plot exhibiting the expression levels of (c) EHMT2-AS1, (d) LINC01018, (e) AL356056.2, (f) AL445524.1, (g) LINC00844, (h) AC093642.1, (i) TRIM52-AS1, (j) AC003991.1, and (k) AC008040.1 between HBV-related HCC and nontumor tissues. lncRNA: long non-coding RNA; HCC: hepatocellular carcinoma.

To assess the optimal diagnostic lncRNA biosignatures for HBV-related HCC, classification models were carried out. The random forest assessment was employed to rank DElncRNAs from large to small, according to the decrease in Mean Decrease Gini as indicated in Figure 2(a). The 10-fold cross-validation indicated that the average accuracy rate of 9 DElncRNAs reached their highest point for the first time as illustrated in Figure 2(b). These 9 DElncRNAs consisting of five upregulated DElncRNAs (AL356056.2, AL445524.1, TRIM52-AS1, AC093642.1, and EHMT2-AS1) and four downregulated DElncRNAs (AC003991.1, AC008040.1, LINC00844, and LINC01018) were identified as the optimal lncRNA molecular markers for the HBV-related HCC diagnosis (Table 1). Therefore, we selected these 9 DElncRNAs as promising optimal lncRNA molecular markers for the diagnosis of HBV-linked HCC, and they were employed to develop the random forest, as well as the SVM models.

### 3.3. Cross-Validation

**Figure 3:**
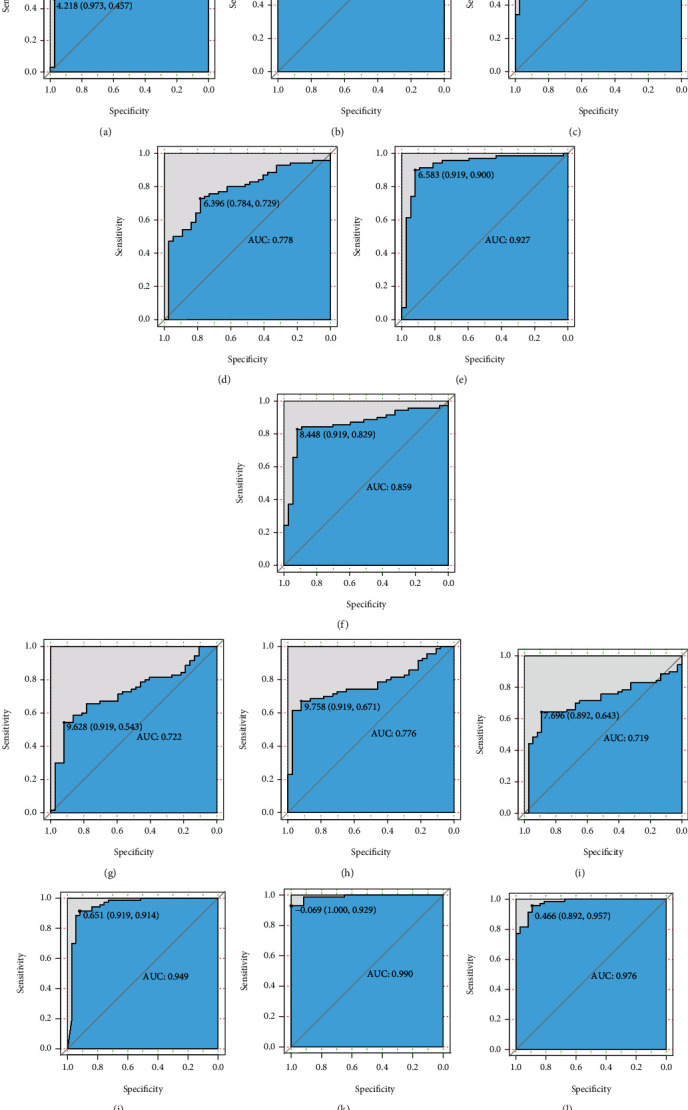
ROC assessment of nine HBV-related HCC-distinct lncRNA biosignatures. The ROC data of these nine diagnostic lncRNA biosignatures (AL356056.2, AL445524.1, TRIM52-AS1, AC093642.1, EHMT2-AS1, AC003991.1, AC008040.1, LINC00844, and LINC01018) and individual (a) AL356056.2, (b) AL445524.1, (c) TRIM52-AS1, (d) AC093642.1, (e) EHMT2-AS1, (f) AC003991.1, (g) AC008040.1, (h) LINC00844, and (i) LINC01018; their combination based on (j) random forest, (k) support vector machine model, and (l) logistic regression. AUC: area under the curve; HCC: hepatocellular carcinoma; ROC: receiver operating characteristic; lncRNA: long noncoding RNA.

A box plot was used to display the levels of expression of the nine DElncRNAs between HBV-linked HCC and nontumor tissues in the validation set (Figures 3(c)–3(g)). The AUC of the random forest model was 0.904, and the specificity and sensitivity were 94.3 and 86.5%, respectively (Figure 3(j)). Moreover, the AUC of the SVM model was 0.957, while the specificity and sensitivity were 95.7 and 100%, respectively (Figure 3(k)). The AUC of the logistic regression model was 0.976, while the specificity and sensitivity were 89.2 and 95.7%, respectively (Figure 3(l)). The ROC of these nine DElncRNAs, AL356056.2, AL445524.1, TRIM52-AS1, AC093642.1, EHMT2-AS1, AC003991.1, AC008040.1, LINC00844, and LINC01018, were 0.750, 0.817, 0.905, 0.778, 0.927, 0.859, 0.722, 0.776, and 0.719, respectively (Figures 3(a)–3(i)), which indicated that these nine lncRNAs were related to HBV-linked HCC, playing a core role in estimating the onset of HBV-linked HCC.

### 3.4. DEmRNA-DElncRNA Coexpressed Analysis

**Figure 4:**
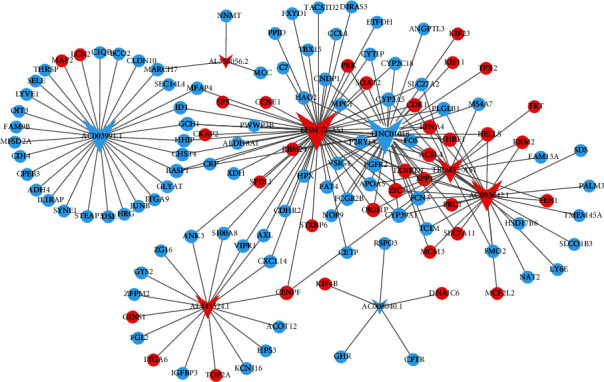
HBV-related HCC-distinct DElncRNA-DEmRNA coexpression network. The V and ellipses indicate the DElncRNAs and DEmRNAs, respectively. Blue and red colors designate downregulation and upregulation, respectively. DEmRNA: differentially expressed mRNA; DElncRNA: differentially expressed long noncoding RNA.

In order to assess the possible functions of these dysregulated lncRNAs in HBV-related HCC, dysregulated DEmRNAs and DElncRNAs were selected to develop a coexpression network. Consequently, the optimal diagnostic lncRNAs were coexpressed with 126 DEmRNAs, accounting for 199 DEmRNA-DElncRNA coexpression pairs. The details pertaining to regulatory information is shown in Figure 4.

### 3.5. Functional and Signal Pathway Enrichment Analysis

**Figure 5:**
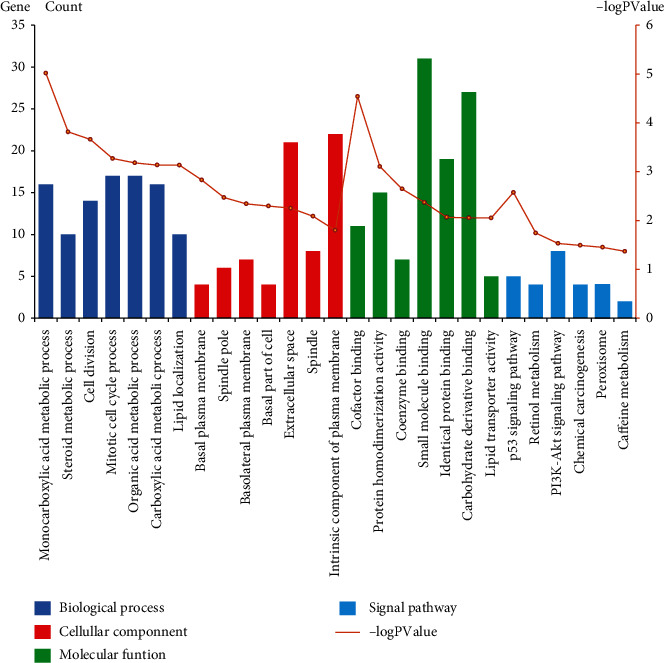
The Gene Ontology along with the signal pathway enrichments.

The above-mentioned coexpression of 126 DEmRNAs with optimal diagnostic lncRNAs was employed to conduct the GO, as well as the KEGG enrichment analyses. GO analysis could classify genes into 3 categories: biological process (BP), cellular component (CC), and molecular function (MF). *P* < 0.05 indicated statistical significance, in which the 3 parts of the GO results of the dysregulated genes are illustrated in Figure 5. The top 15 data generated from the GO enrichment of the dysregulated genes are indicated in Table 2.

KEGG analysis of DEGs demonstrated that they were majorly associated within the p53 signaling cascade, retinol metabolism, PI3K-Akt signaling cascade, chemical carcinogenesis, peroxisome, and caffeine metabolism. The network diagram was then drawn using the Cytoscape software, as shown in Figure 5. A total of 5 DEmRNAs, including STEAP3, CCNE1, CDK1, RRM2, and IGFBP3, were found to be enriched in the p53 signaling pathway. Meanwhile, four DEmRNAs, including CYP3A5, CYP2C18, ADH4, and HSD17B6, were observed to be remarkably enriched in the retinol metabolism pathway. FGFR2, CCNE1, ITGA9, ITGA6, GYS2, EFNA4, GHR, and SPP1 were enriched in the PI3K-Akt signaling pathway, while four DEmRNAs (CYP3A5, CYP2C18, ADH4, and NAT2) were significantly enriched in the chemical carcinogenesis pathway. Furthermore, four DEmRNAs (XDH, HAO2, ACSL4, and SLC27A2) and two DEmRNAs (XDH and NAT2) were enriched in the peroxisome pathway and caffeine metabolism pathway, respectively.

### 3.6. Protein-Protein Interaction (PPI) Network and Module Analyses

**Figure 6:**
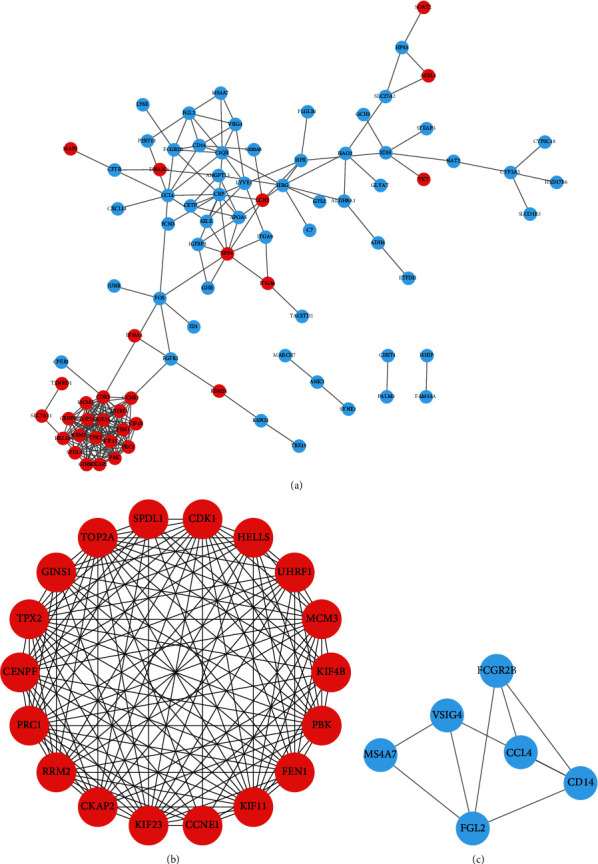
The PPI (protein-protein interaction) network development and significant gene module assessment. Red and blue indicate upregulated and downregulated genes, respectively. (a) PPI networks of DEGs; (b) module 1 consists of 18 nodes/genes; (c) module 2 constitutes of 6 nodes/genes.

Subsequently, a PPI network was built in order to predict the interactions among proteins encoded by the 126 DEGs. As a result, 87 DEGs with a score > 0.4 (medium confidence) were selected for the development of the PPI networks (Figure 6(a)). Afterwards, we clustered two remarkable gene modules by the MCODE APP in Cytoscape, in which module 1 constituted 18 genes/nodes and 127 edges (Figure 6(b)), whereas module 2 comprised 6 genes/nodes and 9 edges (Figure 6(c)). Accordingly, module 1 was primarily abundant in the p53 signaling cascade, cell cycle, and DNA replication, whereas genes in module 2 were primarily abundant in Salmonella infection, NF-kappa B signaling cascade, Toll-like receptor signaling cascade, and phagosome and tuberculosis cascade (Table 3).

## 4. Discussion

Recently, the diagnosis and treatment of hepatocellular carcinoma have been significantly improved. Although HCC patients benefit from a variety of treatment options, the survival time of HCC patients is still limited, regardless of the cancer stage at diagnosis. Hence, in regard to the significance of early diagnosis on survival, further investigations toward the determination of accurate, as well as specific, molecular markers of HCC are urgently needed. A growing number of researches have reported that lncRNA plays a crucial role in hepatocellular carcinoma; lncRNA also has a certain role in HBV-linked HCC [14]. Nevertheless, research on lncRNAs as predictive molecular markers as well as treatment targets remains limited.

Herein, three gene expression datasets were analyzed, in which 38 DElncRNAs along with 543 DEmRNAs were identified in HBV-linked HCC and neighboring samples. The feature selection along with the classification model analysis were employed to identify nine optimal diagnostic lncRNA molecular markers consisting of AL356056.2, AL445524.1, TRIM52-AS1, AC093642.1, EHMT2-AS1, AC003991.1, AC008040.1, LINC00844, and LINC01018 for further analysis. Accordingly, the possible role of these uncovered genes and their correlation with the occurrence and progress of HBV-related HCC were studied.

GO analyses uncovered the prospective biological roles of the abnormally expressed protein-coding genes, while the GO function enrichment results demonstrated that the dysregulated genes were associated with the monocarboxylic acid metabolic and steroid metabolic processes, cell division, mitotic cell cycle process (BP), basal plasma membrane, spindle pole, basolateral plasma membrane, basal part of cell, extracellular space, spindle (CC), cofactor binding, protein homodimerization activity, coenzyme binding, small molecule binding, and identical protein binding (MF). Moreover, the results showed that DEGs were involved in proliferation and the cell cycle of HCC cells induced by chronic hepatitis B.

The pathway analysis demonstrated that the integrated DGEs were particularly abundant in the p53 signaling cascade, retinol metabolism, PI3K-Akt signaling axis, chemical carcinogenesis, and peroxisome. Previous researches have verified that the p53 signaling cascade has a pivotal role in modulating HCC tumorigenesis, mediating HCC inhibition, apoptosis, and senescence, and DNA damage [15]. The PI3K/Akt signaling cascade is overactive in numerous cancers, and activation of this pathway contributes to cell proliferation, survival, and motility, as well as angiogenesis [16, 17]. Previous investigations have demonstrated that the PI3K/Akt signaling cascade is involved in HCC occurrence and metastasis [18]. Cancer is definitely linked to abnormal metabolic processes, and altered retinol metabolism is a pathway that participates in the process of liver fibrosis, where enzymes related to retinol metabolism are linked to liver cancer [19]. Increasing data have suggested that peroxisomes have a pivotal role in cancer. Enzymes involved in peroxisome lipid processing have been previously found to be elevated in a variety of cancers, including liver cancer [20]. Peroxisomes participate in the onset and progress of tumors through autophagic degradation, regulation of cellular immune response, and regulation of metabolism by crosstalk with the mitochondria [21]. Chemical carcinogenesis is a process in which cells undergo genetic and epigenetic variation based on oncogene and tumor suppressors [22, 23]. To this effect, studies have documented that chemical carcinogenesis participates in the onset and progress of liver cancer [24–26]. Therefore, exploring these cascades will aid and elucidate the mechanism of occurrence, proliferation, and invasion of HBV-linked HCC and help predict tumor progression.

The ROC data illustrated the significant diagnostic value of these nine diagnostic lncRNA biosignatures and their combinations for HBV-related HCC. Upregulated EHMT2-AS1 was found to be in the core of the coexpression network, with an AUC of 0.927 and a specificity along with a sensitivity of 90.0 and 91.9%, respectively. Genome-wide association studies also demonstrated that the variants of EHMT2-AS1 were linked to a risk of chronic hepatitis B [27, 28]. It has been reported that upregulated TRIM52-AS1 served an oncogenic role in driving HCC progression, which may be employed as a novel treatment target for HCC [29]. Herein, TRIM52-AS1 was observed to be elevated in HBV-linked HCC tissues in contrast to the neighboring tissues; however, the diagnostic significance of HBV-related HCC was shown to have an AUC of 0.905, as well as a specificity and sensitivity of 80.0 and 94.6%, respectively.

Research has shown TRIM52-AS1 upregulation in both HCC tissue and cell lines. TRIM52-AS1 silencing suppresses cell growth, migration, and infiltration, as well as epithelial-mesenchymal transition *in vitro* while repressing tumor growth *in vivo*. In a certain mechanism, TRIM52-AS1 sponged miR-514a-5p in promoting HCC progression by increasing MRPS18A expression. In contrast, another study demonstrated TRIM52-AS1 downregulation in renal cell carcinoma (RCC). Here, overexpression of TRIM52-AS1 inhibited cell migration as well as proliferation, triggered apoptosis of RCC cells *in vitro*, and interfered with its expression, leading to the opposite effect [30]. However, the underlying biological function of TRIM52-AS1 in cellular processes remains unclear. Further investigation may provide novel insight into the development along with the progression of HBV-linked HCC.

LINC01018 was found to be downregulated in this study. It has been reported that LINC01018 and FOXO1 decreased in HCC, in which LINC01018 sponged miR-182-5p targeted with FOXO1. Overexpression of LINC01018 sponging miR-182-5p repressed proliferation and enhanced HCC cell apoptosis via upregulation of FOXO1 [31]. Moreover, studies have shown that LINC01018 plays critical roles in HBV-induced liver cancer via ceRNA networks [32].

Additionally, investigations have demonstrated that LINC00844 affects drug metabolism and toxicity by regulating the expression of the drug-metabolizing enzyme [33]. Meanwhile, LINC00844 was reported to be downregulated in HCC, and its overexpression remarkably suppressed the proliferation, migration, and invasion of HCC cells [34].

Other than EHMT2-AS1, TRIM52-AS1, LINC01018, and LINC00844, the other five lncRNAs have never previously been documented, and their biological roles remain unclear. The coexpression assessment of lncRNA-mRNA constitute the most frequent approach for determining the prospective target genes of lncRNA and further studying its biological role in many diseases. Specific lncRNA biomarkers of HBV-linked HCC were employed to develop the coexpression network of DElncRNA-DEmRNA, and the role of DEmRNA coexpressed with the nine lncRNAs was annotated.

Accordingly, DEmRNAs and STEAP3 coexpressed with AC003991.1; CCNE1 coexpressed with EHMT2-AS1; CDK1 coexpressed with LINC01018, EHMT2-AS1, and TRIM52-AS1; RRM2 coexpressed with AC093642.1 and TRIM52-AS1; and IGFBP3 coexpressed with AL445524.1 were found to be enriched in the p53 signaling pathway. Cancer cells require considerable amounts of iron due to rapid proliferation; STEAP3 encodes protein functions as an iron transporter, which may regulate intracellular iron storage and help tumors grow in iron-deficient environments [33]. Additionally, STEAP3 and STEAP4 can form a heterodimer, which can affect metal homeostasis and apoptosis, as well as cell cycle regulation [35]. Cyclin E has been implicated in various carcinomas; specifically, CCNE1 (Cyclin E1) is upregulated by HOXC13 to promote proliferation and cell cycle progression [36]. CCNE1 overexpression is linked to the initiation of liver cancer [37] and dismal prognosis in patients suffering from HCC [38]. Moreover, Cyclin-dependent kinase 1 (CDK1) is a vital player in cell cycle modulation, where elevated CDK1 is linked to dismal overall survival in individuals with HCC [38]. CDK1/PDK1/*β*-Catenin cascades are activated in HCC, which may function as a prospective treatment target pathway [39]. RRM2 is a ribonucleotide reductase, a rate-limiting enzyme for cell replication, which catalyzes the reduction of ribonucleotides into deoxyribonucleotides during DNA synthesis. Research has shown that RRM2 modulates cancer cell growth, differentiation, and metastasis, as well as drug resistance, which is overexpressed in prostate cancer, breast cancer, and cervical cancer [40–43]. The IGFBP3 (insulin-like growth factor-binding protein-3) can competitively bind IGF1 (insulin-like growth factor-1) with the IGF1 receptor, thereby modulating cell growth and differentiation, as well as apoptosis. Furthermore, IGFBP3 is decreased in HCC, and low expressions of IGFBP3 are remarkably linked to the tumor size, portal vein invasion, and histological differentiation, as well as capsular invasion [44]. Serum levels of IGF1 along with IGFBP3 are also positively correlated with HCC incidence [45]. IGFBP3 silencing in various tumors is because of the methylation of its promoter, while the high IGFBP3 expression triggers apoptosis and inhibits cell survival, as well as growth [46, 47]. Therefore, the present study puts forward that AC003991.1, EHMT2-AS1, LINC01018, TRIM52-AS1, AC093642.1, and AL445524.1 have pivotal roles in the onset or progress of HBV-related HCC by modulating the p53 signaling cascade.

Cytochrome P450 enzymes participate in multiple types of cancer through a variety of mechanisms, including metabolic enzymes, catalysis of biological activity of chemical carcinogens, activating cancer treatment drugs, and as targets for cancer therapy [48, 49]. CYP3A5, cytochrome P450 family 3 subfamily A member 5, encodes a member of the cytochrome P450 superfamily of enzymes that participate in the metabolic processes of endogenous molecules, such as drugs and steroids, as well as exogenous carcinogens [50]. CYP3A4 along with CYP3A5 account for about 30% of hepatic cytochrome P450, and about half of the drugs oxidatively metabolized by P450 are CYP3A substrates [51]. In recent years, however, numerous studies have documented that CYP3A5 is aberrantly expressed in multiple tumors, and its aberrant expression is strongly related to the infiltration as well as metastasis of tumors [52, 53]. Previous studies have also shown that CYP3A5 suppresses HCC pathogenesis and metastasis through the modulation of the mTORC2/Akt signaling cascade, serving as a prognostic biosignature [54, 55]. Aflatoxin is one of the leading predisposing factors for HCC, and CYP3A5 polymorphism is linked to elevated mutagenic AFB1-exo-8,9-epoxide levels, which may control the individual risk of HCC [56]. In accordance with these findings, the data of the present study demonstrated that CYP3A5 was reduced in HCC. CYP3A5, which coexpressed with LINC01018, EHMT2-AS1, and AC008443.1, was observed to be enriched in both the retinol metabolism and chemical carcinogenesis signaling pathways. Therefore, it was hypothesized that LINC01018, EHMT2-AS1, and AC008443.1 may serve important roles in HBV-related HCC by modulating the expression of CYP3A5 or retinol metabolism and chemical carcinogenesis.

A KEGG pathway evaluation of genes was also conducted in the two most important modules according to the module analysis of the generated PPI network. The results demonstrated that the genes in module 1 were primarily abundant in the p53 signaling cascade, cell cycle, and DNA replication, whereas the genes in module 2 were primarily abundant in the Salmonella infection, NF-kappa B signaling axis, Toll-like receptor signaling cascade, and phagosome, as well as tuberculosis pathways. These data suggested that the onset along with the development of HBV-linked HCC may be correlated to these cascades, and the regulation of cell cycle-related biological processes or pathways may be an effective treatment for HBV-related HCC.

## 5. Conclusion

Here, a 9-lncRNA diagnosis model was constructed based on microarray datasets to highlight its diagnostic significance for HBV-linked HCC. In this regard, the functional annotation of DEmRNAs coexpression with nine lncRNA molecular markers of HBV-related HCC yielded valuable insights in assessing the pathogenesis of HBV-related HCC. Collectively, these data may provide an understanding of the regulatory mechanisms of lncRNAs in the onset, as well as the development of HBV-related HCC, and help identify potential diagnostic and treatment targets for HBV-linked HCC patients. Nevertheless, this work had some limitations. The sample size was small, and the samples were acquired from an independent patient cohort. Furthermore, this study only preliminarily predicts the role of lncRNAs in HBV-linked HCC, and further wet experiments are required in order to verify their mechanisms.

## Figures and Tables

**Table 1:** Nine optional diagnostic lncRNA biomarkers for HBV-related HCC.

lncRNA	LogFC	P value	Regulation
EHMT2-AS1 AL356056.2 TRIM52-AS1 AC093642.1 AL445524.1 AC003991.1 LINC01018 LINC00844 AC008040.1	1.220 1.104 1.292 1.413 1.252 -2.345 -1.713 -2.331 -1.329	0.01012 0.01372 0.02182 0.03369 0.04125 9.16E-05 0.00033 0.00097 0.00451	Up Up Up Up Up Down Down Down Down

**Table 2:** Top 15 GO enrichment terms associated with the dysregulated genes.

Category	Term	Count	P value
BP BP BP BP BP CC CC CC CC CC MF MF MF MF MF	Monocarboxylic acid metabolic process Steroid metabolic process Cell division Mitotic cell cycle process Organic acid metabolic process Basal plasma membrane Spindle pole Basolateral plasma membrane Basal part of cell Extracellular space Cofactor binding Protein homodimerization activity Coenzyme binding Small molecule binding Identical protein binding	16 10 14 17 17 4 6 7 4 21 11 15 7 31 19	9.59*E*-06 1.53*E*-04 2.19*E*-04 5.39*E*-04 6.61*E*-04 1.48*E*-03 3.39*E*-03 4.55*E*-03 5.06*E*-03 5.59*E*-03 2.88*E*-05 7.87*E*-04 2.26*E*-03 4.27*E*-03 8.58*E*-03

**Table 3:** KEGG pathway enrichment of modular genes in protein interaction network.

Module	Term	Count	P value	Genes
Module 1	p53 signaling pathway	3	8.96*E*-04	CCNE1, CDK1, RRM2
	Cell cycle	3	0.003177	CCNE1, CDK1, MCM3
	DNA replication	2	0.026391	MCM3, FEN1
Module 2	Salmonella infection	2	0.023007	CCL4, CD14
	NF-kappa B signaling pathway	2	0.025265	CCL4, CD14
	Toll-like receptor signaling pathway	2	0.028646	CCL4, CD14
	Phagosome	2	0.040436	FCGR2B, CD14
	Tuberculosis	2	0.049092	FCGR2B, CD14

## Data Availability

The datasets, as well as the code generated or analyzed in this work, can be accessed from the corresponding authors on reasonable request.

## Supplementary Materials

Supplementary Materials PCA analysis of quality control over three datasets used in this study.
