# Supplementary figures and images for: Potential lncRNA Biomarkers for HBV-Related Hepatocellular Carcinoma Diagnosis Revealed by Analysis on Coexpression Network
Source: Biomed Res Int. 2021 Oct 15;2021:9972011. doi: 10.1155/2021/9972011 (PMC8536424; doi:10.1155/2021/9972011)

# Supplementary Figure

GSE84402

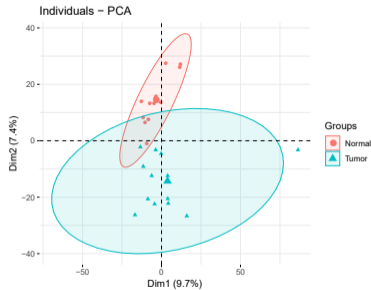

GSE55092

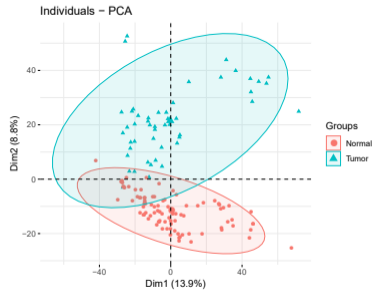

GSE19665

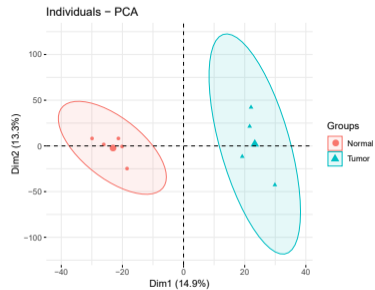

Supplement: Supplementary Materials — PCA analysis of quality control over three datasets used in this study. [file 9972011.f1.pdf]
